# Supplementary figures and images for: Distinct Fatty Acid Compositions of HDL Phospholipids Are Characteristic of Metabolic Syndrome and Premature Coronary Heart Disease—Family Study
Source: Int J Mol Sci. 2021 May 6;22(9):4908. doi: 10.3390/ijms22094908 (PMC8124224; doi:10.3390/ijms22094908)

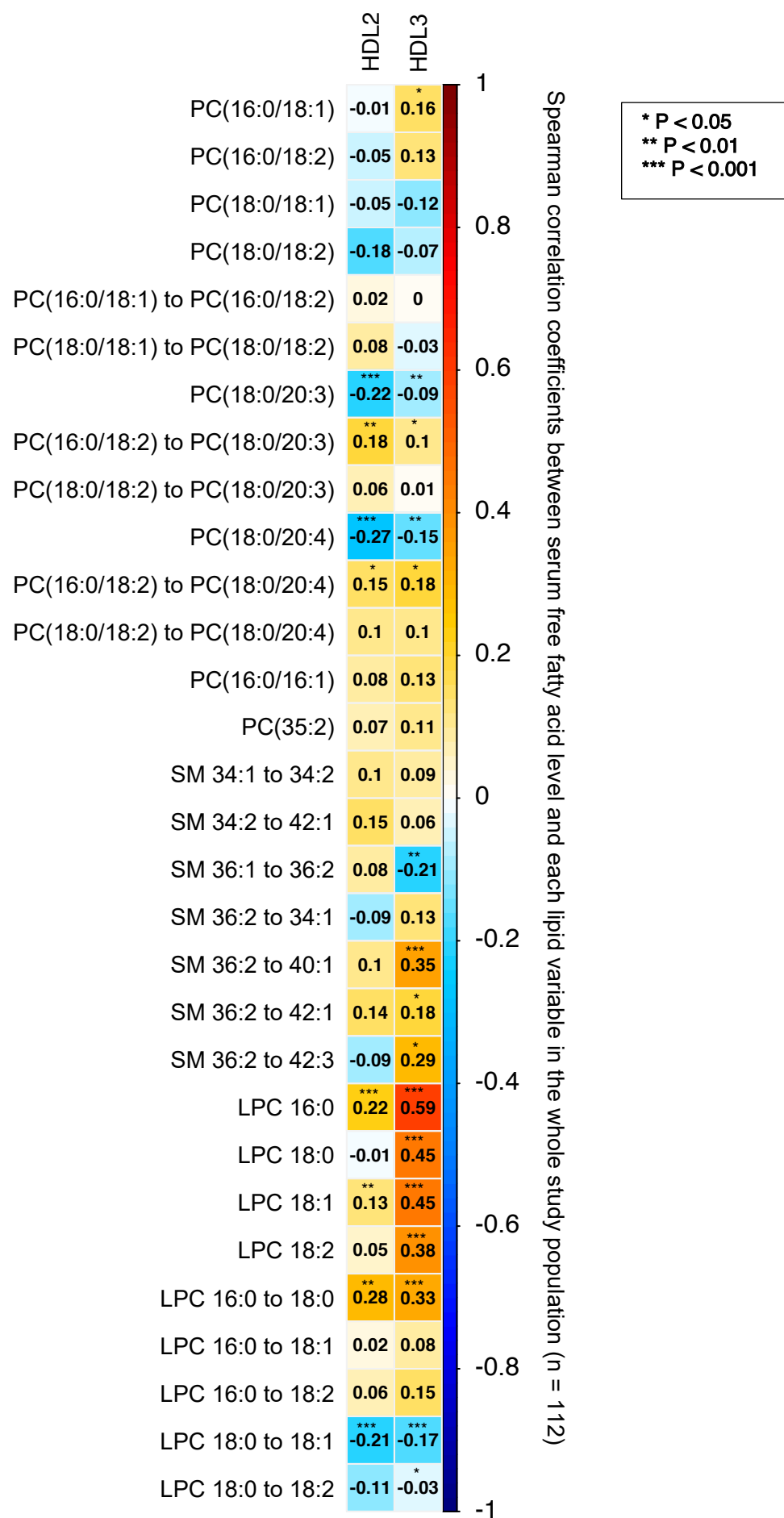

Supplement: Supplementary file 1 [file ijms-22-04908-s001.zip › Figure S1.pdf]
